# Deep Learning-Based Computer-Aided Pneumothorax Detection Using Chest X-ray Images

**DOI:** 10.3390/s22062278

**Published:** 2022-03-15

**Authors:** Priyanka Malhotra, Sheifali Gupta, Deepika Koundal, Atef Zaguia, Manjit Kaur, Heung-No Lee

**Affiliations:** 1 Chitkara University Institute of Engineering and Technology, Chitkara University, Patiala 140401, Punjab, India; priyanka.malhotra@chitkara.edu.in (P.M.); sheifali.gupta@chitkara.edu.in (S.G.); 2 Department of Systemics, School of Computer Science, University of Petroleum and Energy Studies, Dehradun 248007, Uttarakhand, India; dkoundal@ddn.upes.ac.in; 3 Department of Computer Science, College of Computers and Information Technology, Taif University, P.O. Box 11099, Taif 21944, Saudi Arabia; zaguia.atef@tu.edu.sa; 4 School of Electrical Engineering and Computer Science, Gwangju Institute of Science and Technology, Gwangju 61005, Korea; manjitkaur@gist.ac.kr

**Keywords:** deep learning, medical imaging, mask RCNN, image segmentation, pneumothorax

## Abstract

Pneumothorax is a thoracic disease leading to failure of the respiratory system, cardiac arrest, or in extreme cases, death. Chest X-ray (CXR) imaging is the primary diagnostic imaging technique for the diagnosis of pneumothorax. A computerized diagnosis system can detect pneumothorax in chest radiographic images, which provide substantial benefits in disease diagnosis. In the present work, a deep learning neural network model is proposed to detect the regions of pneumothoraces in the chest X-ray images. The model incorporates a Mask Regional Convolutional Neural Network (Mask RCNN) framework and transfer learning with ResNet101 as a backbone feature pyramid network (FPN). The proposed model was trained on a pneumothorax dataset prepared by the Society for Imaging Informatics in Medicine in association with American college of Radiology (SIIM-ACR). The present work compares the operation of the proposed MRCNN model based on ResNet101 as an FPN with the conventional model based on ResNet50 as an FPN. The proposed model had lower class loss, bounding box loss, and mask loss as compared to the conventional model based on ResNet50 as an FPN. Both models were simulated with a learning rate of 0.0004 and 0.0006 with 10 and 12 epochs, respectively.

## 1. Introduction

Pneumothorax is a thoracic disease condition in which the lungs of a human being collapse, causing air to leak into the pleural cavity, which is the area surrounding the lungs and the walls of the chest. The leaked air then pushes the outside boundary of the lung and results in the collapse of lungs. This may be a complete collapse of the lungs or a collapse of just one part. 

Pneumothorax can occur due to an injury to the chest which causes a tear on the lung surface, allowing air to get trapped in the pleural cavity, due to some underlying lung diseases such as pneumonia, chronic obstructive pulmonary disease (COPD), etc., or if the air trapped in the pleural cavity does not escape and continues to grow [1,2]. A person suffering from pneumothorax may have sudden pain in the chest or difficulty with breathing. Pneumothorax can be life-threatening, as it can lead to cardiac arrest, failure of the respiratory system, or, in extreme cases, even death. As per [3,4], there are 99.9 cases of spontaneous pneumothorax per 100,000 hospital admissions annually. According to Martinelli et al. in [5], Pneumothorax has been identified as one of the important factors complicating the cases of the coronavirus disease COVID-19 and increasing the rate of hospital admission. The proper diagnosis and medication is important to increase the survival rate and prevent any life threat caused by this disorder. It is difficult to diagnose pneumothrax by physical examination of a patient.

Chest radiographic images or chest X-ray (CXR) imaging is the primary diagnostic imaging technique employed in the diagnosis of pneumothorax, as it provides a quick diagnosis. The interpretation of chest radiographic images for diagnosing pneumothorax is difficult [6,7]; images may have some superimposed structures, patterns of different thoracic diseases has diverse appearances, sizes, and locations on CXR images, and the varying postures of patients while capturing the X-ray image can create distortion. In addition, the accurate pixel-level annotations in CXR can be done by highly experienced radiologists, resulting in high expenses. The experienced radiologists are not easily available in undeveloped areas. In [8], the author identified a shortage of expert radiologists who can detect the presence of an abnormality from a chest X-ray, even when the X-ray equipment is available. This has created an interest in computerized diagnosis of pneumothorax from chest radiographic images. A computerized diagnosis system can detect pneumothorax in chest radiographic images which provide substantial benefit in disease diagnosis.

In the last few years, a computerized diagnosis of disease using artificial intelligence (AI) has emerged as a major research topic in the area of medical diagnosis. AI systems can improve the performance of any disease diagnosis system by minimizing the number of errors during the interpretation of the image [9]. The deep learning model has been significant in the medical image analysis field. The use of deep learning algorithms has led to development in the field of biomedical image analysis. The new deep learning model has been developed for the task of classification and segmentation of medical images for presence of disease.

Image segmentation is a process of partitioning a given digital image into different segments. The pixels in the image with similar attributes are grouped together. Image segmentation [10] is classified into two categories: semantic segmentation and instance segmentation. Semantic segmentation is a method of assigning labels to all the pixels in an image such that the pixels connected to each other by certain properties belong to the same label. Instance segmentation involves partitioning of boundaries of individual objects in an image at pixel level. In [11], the authors stated that instance segmentation detects and delineates each object of interest in the image. The segmentation of lesions in medical images can aid in monitoring the geometric changes in the size of lesions and in calculating dosage of medicine. The use of deep neural networks can aid in improving the health care system and providing access to detection of disease in the absence of chest radiograph experts.

In the present work, a deep learning model is proposed for segmenting regions with traces of pneumothorax in chest X-ray images. The proposed model uses Mask RCNN with ResNet101 as a backbone feature pyramid network. The model has been trained utilizing transfer learning by using pretrained weights of pneumonia identification algorithm [12]. The model was trained on a SIIM-ACR pneumothorax segmentation challenge dataset which is available on Kaggle and can be accessed at: https://www.kaggle.com/c/siim-acr-pneumothorax-segmentation (accessed on 18 January 2022).

The major contributions of this study are as follows:

(i) SIIM-ACR pneumothorax segmentation dataset has been preprocessed using data augmentation and upsampling techniques.
(ii) An MRCNN model based on ResNet101 as a backbone feature pyramid network (FPN) is proposed to detect the areas of pneumothorax in chest X-ray images.
(iii) The performance of the proposed neural network model with ResNet101 FPN was analyzed and compared with the conventional model using ResNet50 as FPN.
(iv) The performance of the proposed neural network model was compared with the existing models.

The rest of the paper is organized as follows: Section 2 explains the related research in the area of deep-learning-based medical image segmentation. Section 3 presents the dataset used for training the proposed model. Section 4 discusses the architecture of the proposed model. Section 5 describes the workflow of the proposed model. The result analysis of the proposed model is conferred in Section 6. Section 7 concludes the present research work and gives future scope.

## 2. Related Research

A deep learning model has been extensively employed in the field of medical image analysis for classification and segmentation of diseases. The classification of medical images can be done using various deep learning models. As compared with classification, the techniques for localization of abnormalities in medical images give more information regarding disease diagnosis and probabilistic prognosis. DL-based image segmentation models can predict the label for each pixel in the image [13]. The authors in [14] presented a fully automated framework employing 2D and 3D CNN to segment cardiac MR images. In [15], the authors introduced a recurrent neural network (RCN) architecture to perform segmentation of the pancreas in abdominal MRI and CT images. The model design consisted of a deep convolutional subnetwork with the output layer connected to a long short term memory (LSTM) network. In [16], the authors reported a 3D deep residual network for volumetric segmentation of the brain in MR images. Authors in [17] proposed a cascaded FCN model to segment the liver and the lesions within the ROI. A dense 3D conditional random field was employed to produce final segmentation. In [18], the authors proposed a 3D deeply supervised network (DSN) with fully convolutional architecture for automatic segmentation of the liver in CT images. The designed model attained fast convergence and good discrimination capability on the MICCAI-Sliver07 (Medical Image computing and computer assisted intervention) dataset. Dhungel et al. [19] reported a deep convolution and deep belief network for segmenting breast masses in Mammography images. The authors employed two different loss minimization parameter learning algorithms, CRF and structured SVM, with CRF being faster. Poudel et al. [20] developed a recurrent fully convolutional network (RFCN) to detect and segment the heart in cardiac MR images. Hamidian et al. in [21] converted 3D CNN into 3D FCN to segment pulmonary nodules in chest CT images. In [22], Stollenga et al. suggested a recurrent neural network taking advantage of multidimensional LSTM for pixel-wise segmentation of MR images of the brain. In [23], Zhang et al. proposed a model with the dilated and separable convolution into residual U-Net architecture for segmenting brain tumors in MR images. Milletari et al. [24] employed a V-Net model to segment the prostate in MRI images. Mulay et al. [25] suggested a nested edge detection and Mask RCNN network for segmentation of the liver in CT and MR images. Gordienko et al. [26] reported a U-Net based CNN for segmentation of the lungs on CXRs images.

The DL-based segmentation techniques can be utilized for locating abnormalities in chest radiographic images. In [27], GooBee et al. proposed three different networks, namely CNN, FCN, and MIL (multi instance learning), for classification and localization of pneumothorax in chest X-ray images. In [28], Taylor et al. suggested a deep convolutional network to identify pneumothorax in the chest X-ray dataset. In [29], authors designed a CheXLocNet algorithm based on Mask R-CNN to segment the area of pneumothorax from chest radiographs. The authors employed Mask RCNN with ResNet-50 as a backbone feature pyramid network. In [30], the authors proposed a two-stage U-Net model with ResNet 34 as a backbone neural network for segmentation of pneumothorax. The authors concluded that two-stage training of U-Net showed better network convergence. In [31], the authors suggested a design consisting of an ensemble of three LinkNet networks with se-resnext50, se-resnext101, and SENet154.In the present work, a mask regional convolutional neural network (MRCNN) model with ResNet101 as a backbone feature pyramid network has been proposed for segmentation of regions containing pneumothorax in chest X-ray images.

## 3. Dataset Analysis

The Society for Imaging Informatics in Medicine, in collaboration with American College of Radiology (SIIM-ACR), collected the CXR data for pneumothorax and released it on Kaggle. The SIIM-ACR dataset was used for training, validation, and testing of the proposed model, and is available at: https://www.kaggle.com/c/siim-acr-pneumothorax-segmentation (accessed on 18 January 2022). The dataset contained three files: DICOM training images, DICOM testing images, and run-length encoded files.

**Figure 1:**
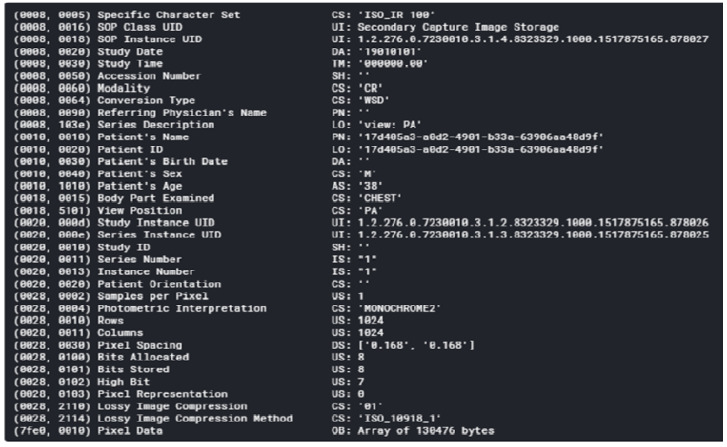
Snapshot of metadata stored in a DICOM Image.

A DICOM (Digital Imaging and Communications in Medicine) format consists of header data and an image, both of which are packed into a single file. The header of the DICOM file consists of a series of tags that provide information concerning the patient’s name, age, sex, demographics, and various other parameters (as shown in Figure 1). Important information regarding the patient can be extracted from these tags. The images in the DICOM files contained either frontal AP (anterior–posterior) or frontal PA (posterior–anterior) chest radiographs for a particular patient.

**Figure 2:**
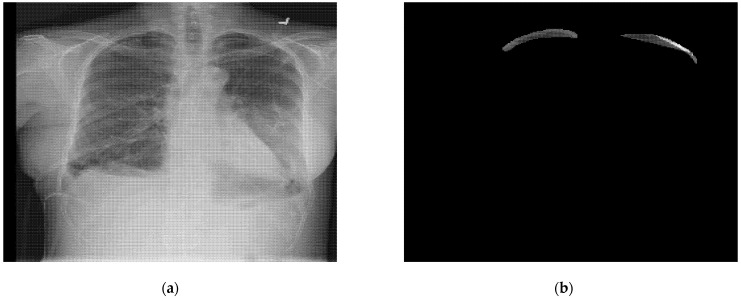
A training image from the dataset; (**a**) chest X-ray DICOM image (**b**) segmentation mask obtained from RLE file.

The dataset consisted of 12,052 images in DICOM format that were 1024 × 1024 pixels. There were around 10,675 training images and 1377 testing images. The training and testing images were stored in separate folders, and the images had a .dcm extension. A DICOM training image from the dataset with a .dcm extension is shown in Figure 2a.

The run-length-encoded files were in the form of an excel file with .csv extension, storing the annotations mask for the dataset images. These excel files had the data in the form of run-length-encoded (RLE) code. The RLE file contained two columns: image ID, indicating the image number, and the encoded pixel column, indicating the pixel numbers with mask values for the given image ID. The RLE code was decoded to generate the segmentation mask. The segmentation mask obtained from the RLE file is shown in Figure 2b.

## 4. Architecture of Proposed Mask RCNN Model

**Figure 3:**
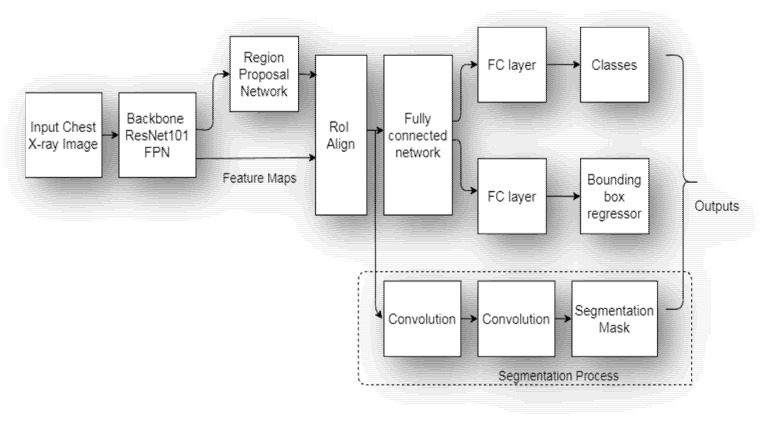
Proposed Mask RCNN Model Architecture.

The segmentation model proposed in the present work is based on the Mask Regional Convolutional Neural Network [32] with ResNet101 [33] as a backbone FPN. Mask RCNN is a deep neural network model that generates bounding boxes as well as segmentation masks for every instance of an object present in the given image. The architecture of the proposed model is shown in Figure 3.

### 4.1. Backbone ResNet101 Feature Pyramid Network (FPN)

**Figure 4:**
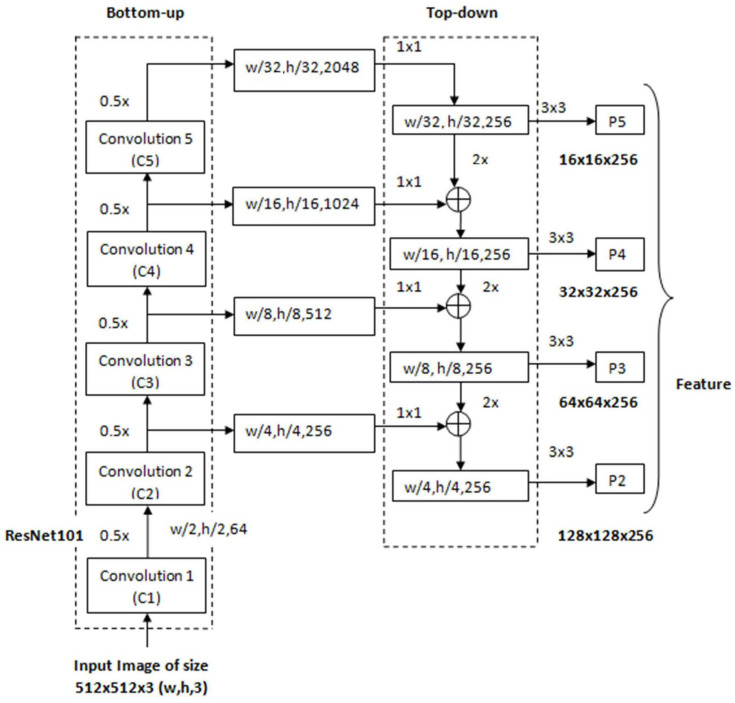
ResNet101-based Feature Pyramid Network Architecture.

The backbone deep neural network called the feature pyramid network is used to extracting features. It consists of three parts: the bottom-up pathway, top-down pathway, and lateral connections (shown in Figure 4). The bottom-up pathway of the proposed model consists of ResNet101 [34] for extracting features from the input image. The proposed model is different from the existing model [29] in terms of the backbone network. In the proposed model, ResNet101 has been used as a backbone network, whereas in the existing model [29], ResNet50 has been used. The ResNet101 is different from ResNet50 in terms of the number of layers, as depicted in Table 1. The bottom-up pathway has one pyramid level for each of the stages. The bottom-up pathway extracts the feature map from the input image. These feature maps undergo 1 × 1 convolutions for channel dimensionality reduction. The output of the bottom-up pathway acts as a reference feature map for the top-down pathway by a lateral connection.

The feature maps from the two pathways are merged and use element-wise addition. A 3 × 3 convolution is applied to each merged feature map to generate the final feature map. The final set of feature maps generated by the FPN, termed {P2, P3, P4, and P5}, has the same spatial sizes [35]. The use of the ResNet-101 FPN backbone improves the accuracy and speed of the proposed model.

In the present work, the different layer of the proposed model was not trained from scratch; the concept of transfer learning has been employed. Transfer learning [36] is a powerful approach in which a model trained for one task can be utilized to initialize the parameters of a model to be trained for another task. Transfer learning is a means for faster and better training of the model with the limited amount of data. In the present work, the weights of our backbone ResNet 101 model were initialized to the weights pretrained on a pneumonia detection challenge. This improved the accuracy and saved model training time.

### 4.2. Regional Proposal Network

**Figure 5:**
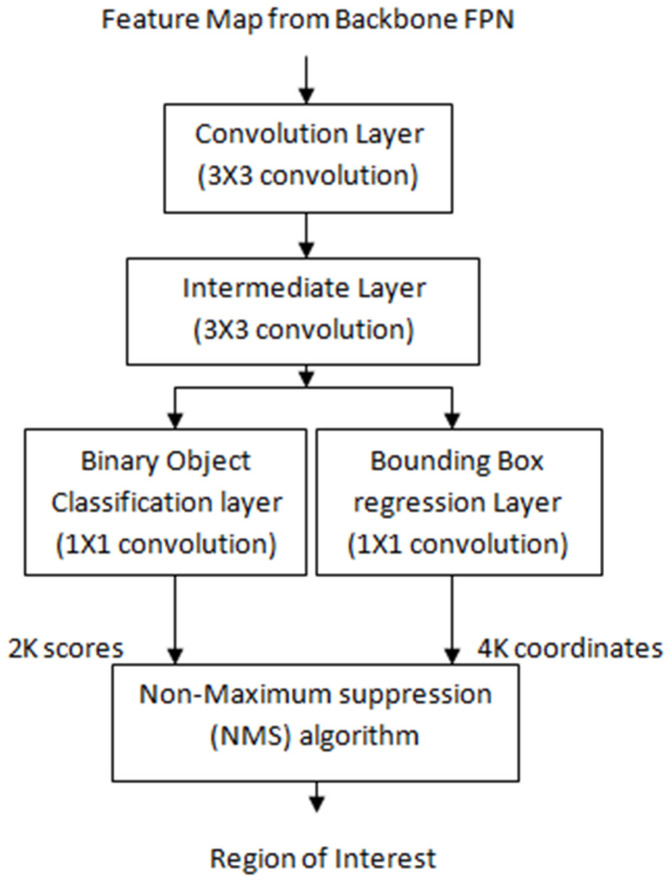
Regional Proposal Network.

**Figure 6:**
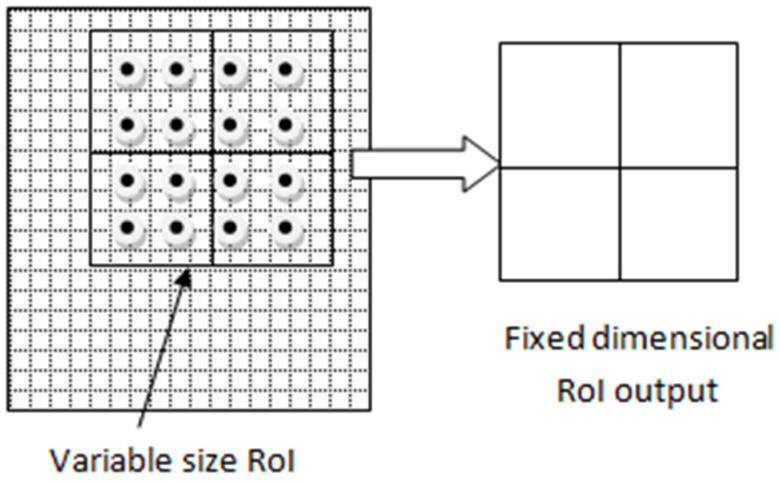
RoI alignment operation.

A regional proposal network (RPN) scans feature maps generated by a backbone network and proposes the Region of Interest or RoI. The RPN creates the bounding boxes called anchor boxes of different sizes and aspect ratios that stretch across the entire input feature map [37]. Researchers have employed different techniques to compute the bounding boxes [38,39]. In the present work, the RPN works as follows:

(i) Anchor generation: A sliding window convolution of 3 × 3 (with 512 filters and padding = same) is applied to the feature maps obtained from the backbone feature pyramid network. The center point of the sliding window represents an anchor. In the proposed model, anchor boxes have a scale of {32^2^, 64^2^, 128^2^, 256^2^} pixels with anchor ratios of {1:2, 1:1, 2:1}. Each sliding window of RPN generates K = 12 anchor boxes with four scales and three aspect ratios. For the entire image, N = W × H × K anchor boxes are generated with W*H being the size of input convolution feature maps. Figure 5 shows the process of the anchor generation.
(ii) Classification scores and bounding box coordinates generation: The anchor or bounding boxes generated in the previous step are passed to an intermediate layer of 3 × 3 convolution (with padding of one) and 256 output channels. As depicted in Figure 6, the output is then passed to two layers of 1 × 1 convolution: the classification layer and regression layer. The classification layer generates a matrix of size (W, H, k × 2) for N anchor boxes with two scores corresponding to the probability of an object existing or not. The regression layer generates a matrix of size (W, H, k × 4) for N anchor boxes with four values of the coordinates of each bounding box (see Figure 5).
(iii) Non maximum suppression (NMS) algorithm: Out of the generated bounding boxes, the best bounding boxes were selected using the non maximum suppression (NMS) algorithm given below:
  (a) Sort all of the created bounding boxes in decreasing order of their object score confidence;
  (b) Select the box with the highest object score confidence;
  (c) Calculate the overlap or intersection over union (IoU) of the current box with the other boxes that belong to the same object class;
  (d) Remove all the boxes with IoU values greater than 0.7;
  (e) Move to the next highest object score confidence;
  (f) Repeat the above steps for all the boxes in the list.

The selected parameters of the RPN for the proposed network are summarized in Table 2.

### 4.3. Region of Interest (RoI) Align

The bounding boxes or region proposals generated by RPN have different scales, and these different scale features are to be sent to a fully connected layer with a fixed scale [40]. RoI align predicts the region of interest from the bounding boxes and uses bilinear interpolation to generate fixed size, 7 × 7 feature maps. The following steps are taken in the RoI align process:

(a) The region proposal candidates are generated by RPN. These region proposal coordinates are floating point numbers, and their boundaries are not quantized.
(b) The region proposal candidate boxes are divided evenly into a fixed number of smaller regions.
(c) In each smaller region, four points are sampled.
(d) The feature pixel values for each point are calculated using bilinear interpolation.
(e) The max-pooling operation is performed on each subregion to obtain the final feature map.

The RoI alignment operation [41] is shown in Figure 6, in which the background grid represents the feature map. The grid is divided into squares, and dots in this grid represent the sample points in a 2 × 2 bin. The bilinear interpolation was applied to these points and a fixed-size (7 × 7) feature map was generated. These fixed-size feature maps were reshaped into a one-dimensional vector by a fully connected network. They further consists of two fully connected layers of size 1024 to classify and predict RoIs category and bounding box.

### 4.4. Segmentation Process

Mask RCNN uses convolution-based neural networks to extract masks for each RoI and segments the image pixel wise [41]. This branch generates a fixed mxm size mask for each class with Km^2^ dimensional output for each of the RoIs with K different classes. In our study, a 28 × 28 mask was generated for each of the regions. During the model training, the ground truth mask contained in the training dataset was downscaled to compute the value of loss with the predicted mask. During the inference, the generated mask was up-scaled to the original size of the ROI bounding box.

## 5. Workflow of Proposed Model

**Figure 7:**
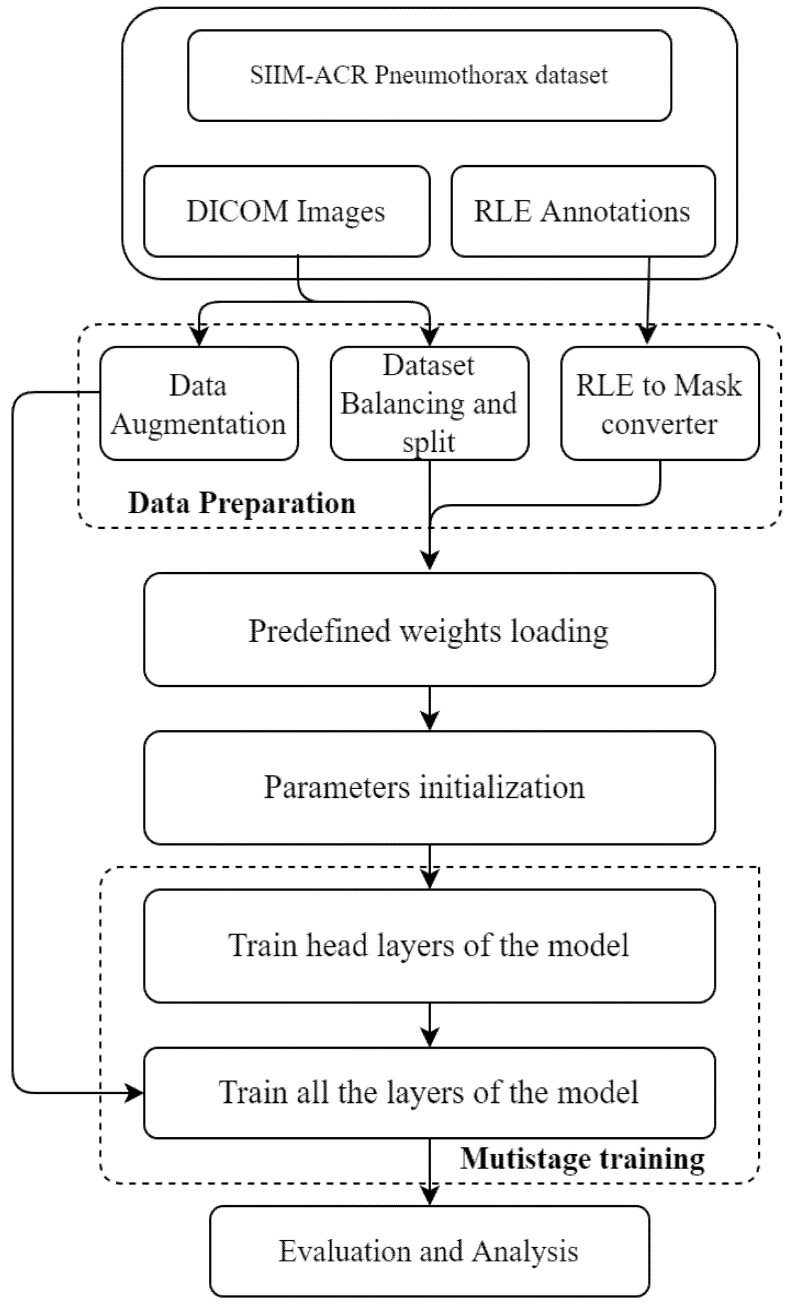
Workflow of the Proposed Model.

The workflow of the proposed model is represented in Figure 7.

The proposed Mask RCNN model with a backbone ResNet101 as an FPN is trained on a SIIM-ACR pneumothorax dataset available on Kaggle. The model is implemented as explained next.

### 5.1. Data Preparation

The SIIM-ACR pneumothorax dataset was downloaded from www.kaggle.com (accessed on 18 January 2022). The dataset consisted of three files containing DICOM training images, DICOM testing images and excel file with mask information encoded using run-length encoding. The operations performed on dataset as explained below:

#### 5.1.1. Data Augmentation

**Figure 8:**
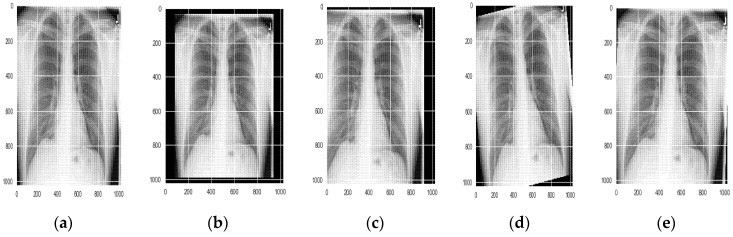
Geometric transformations applied to the image; (**a**) original image (**b**) scaled image (**c**) translated image (**d**) rotated image (**e**) sheared image.

Data augmentation [42] is a technique employed on the training dataset to improve the performance of the deep learning model. These techniques increase the ability of the model to generalize. In the present work, different augmentation techniques were applied to the dataset. The different linear geometric transformation applied includes scaling, the image can be scaled outward or inward; translation, involving moving the image along the X or Y direction (or both); rotation, which rotates the image by a specified degree right or left on an axis (between 1° and 359°); and shearing, which transforms the orientation of the image and shifts one part of the image, similar to a parallelogram. The images resulted as shown in Figure 8.

**Figure 9:**
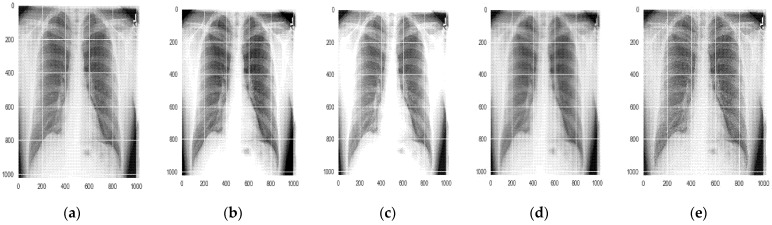
Augmentation applied to images; (**a**) original image (**b**) contrasted image (**c**) multiplied image (**d**) blurred image (**e**) sharpened image.

The other augmentation techniques (see Figure 9) applied to the dataset include multiplication, which multiplies all pixels in an image by a random value sampled uniformly from the interval [0.9, 1.1]; Gaussian blur, which is obtained by blurring an image using a Gaussian function to reduce the noise level; contrast, which gives the degree of separation between the darkest and brightest areas of an image; and sharpening, which highlights edges and fine details in an image.

#### 5.1.2. Dataset Balancing and Splitting

The dataset consisted of 12,052 images in DICOM format with the size of 1024 × 1024 pixels. These images were resized to 512 × 512 pixels. There were around 10,675 training images and 1377 test images. The dataset had high class imbalance and consisted of only 22% positive pneumothorax cases. The number of positive samples in the training set was increased to 53.2% by over-sampling the positive images. The training dataset was further split into two parts: a training and validation dataset. The total numbers of images in the training, validation, and testing datasets after the split are given in Table 3.

#### 5.1.3. RLE to Mask Conversion

**Figure 10:**
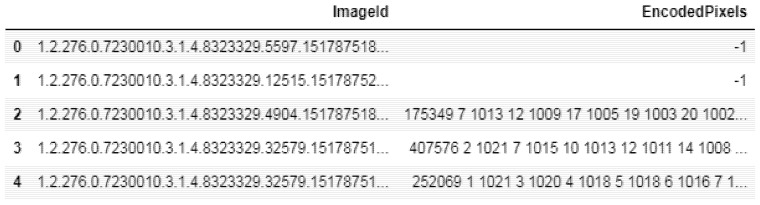
RLE file data for five images.

The annotation mask for the training data was stored in the run-length-encoded (RLE) file with a .csv extension. RLE is a lossless compression method that replaces data sequences having identical values (run) with the respective value stored once, and the length of the run. The RLE file contained two columns, image ID and encoded pixels, for each figure. In Figure 10, image ID and encoded pixels are shown for five images.

The image ID provides the image number. The encoded pixel column marked as −1 indicates that there is no mask for the given image ID. In Figure 10, images no. zero and one have encoded pixel values of −1. This means that there is no mask given for these images due to absence of pneumothorax. The encoded pixels column has values in run-length-encoded form to generate the mask with pneumothorax. In the generated mask, the pixel value is zero for non pneumothorax regions and one for pneumothorax regions.

**Figure 11:**
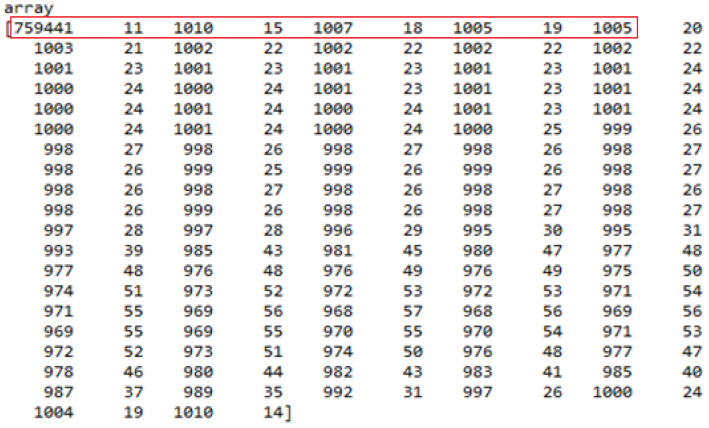
Complete RLE array for reference image ID (red box shows the values utilized for explanation in Table 4.)

The complete RLE array for one reference image ID is shown in Figure 11. The reference image is an array with a size of 1024 × 1024 pixels, having a total number of 1024 × 1024 = 1,048,576 pixels in the form of a vector. For the reference image, the initial pixel position of the mask in the vector is 759,441, where its value is one. After that, 11 consecutive pixels have a value of one. Then, 1010 pixels consecutively have a pixel value of zero. The next pixel position having value a value of one is 759,441 + 11 + 1010 = 760,462. Some of the initial pixel positions and the final pixel positions for pixel values zero and one are shown in Table 4. In this way, the complete mask could be generated for all the pixel positions in the form of a vector. Then, the complete vector, with a size of 1,048,576 pixels, was again converted into an array of size 1024 × 1024.

**Figure 12:**
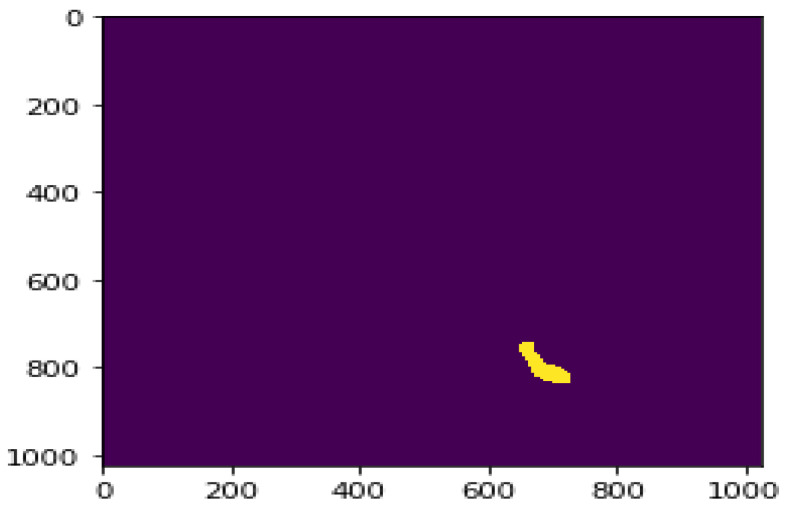
Mask generated for the reference image ID (The yellow color area indicates the mask).

The pixel locations from 759,441 to 759,452, 760,462 to 760,477, 761,484 to 761,502, and 762,507 to 762,526 had a value of one. The same process was applied to all the values stored in the array, and the pixel locations having a value of one were decoded. This process of conversion generated the mask. The generated mask for the reference image ID is given in Figure 12.

### 5.2. Predefined Weights Loading

The proposed model uses pretrained weights from a past medical imaging algorithm used for pneumonia identification, available in [43]. For this, initially Matterport’s Mask RCNN model was installed from github using the command: !git clone Mask_RCNN. Transfer learning was used to train the model [44]. The pretrained weights from pneumonia identification were used as initial parameters for the model and were downloaded with the help of the command: wget--quietmask_rcnn_coco.h5. The use of transfer learning saved the computational expense that would otherwise manifest while training the network from scratch.

### 5.3. Parameter Initialization

In the proposed model, different simulation parameters were initialized. The model was simulated with a backbone as ResNet101. The details regarding the values of the experimental parameters such as number of classes, image dimension, RPN parameters, batch size, epochs, learning momentum, weight decay, etc., are given in Table 5.

### 5.4. Multistage Training

The proposed model was trained on a training dataset consisting of 15,629 images. The proposed model was trained in two stages. In stage 1, the model head layers were trained for one epoch with the learning rate doubled, and no data augmentation was utilized. In stage 2, all the layers of the selected model were trained. The model was simulated for two different learning rates, LR 0.0006 and 0.0004. Similarly, the proposed model was simulated by taking two different values of epochs, i.e., 10 and 12, in stage 2. Each epoch consisted of 350 iterations. Table 6 represents the simulation parameters for stages 1 and 2, respectively.

## 6. Results and Discussion

Python has emerged as one of the most simple and efficient languages for implementing deep learning algorithms. It is used in various image classification and segmentation tasks. The code for the present work was written in Python. The code was run on the NVIDIA Tesla P100 GPU. The following important libraries of Python were utilized for developing the proposed model: Keras, Tensorflow, openCV, pydicom, imaug, h5py, and scikit-image.

### 6.1. Results for Segmentation of Pneumothorax

**Figure 13:**
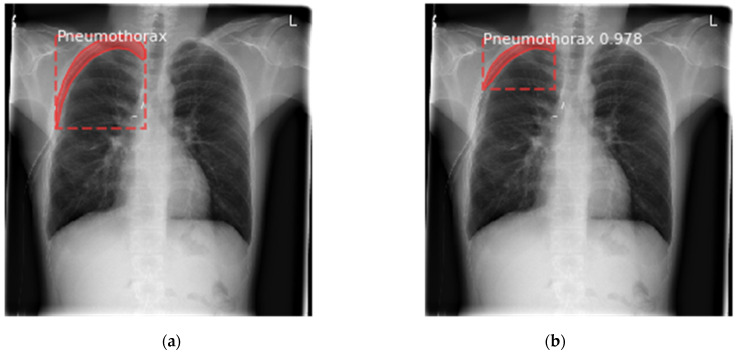
Sample validation dataset image with different annotations predicted by the proposed model, (**a**) annotations on ground truth image; (**b**) annotations predicted by proposed model.

The proposed Mask RCNN model draws the dotted bounding box around each detected region of pneumothorax. Further, it assigns class labels for each detected region witha prediction confidence score. Moreover, it creates the object mask for each of the pneumothorax regions. The image shown in Figure 13 depicts the different annotations generated on a sample taken from the validation dataset. The proposed model generated the segmentation mask and predicted the confidence score for each image efficiently.

**Figure 14:**
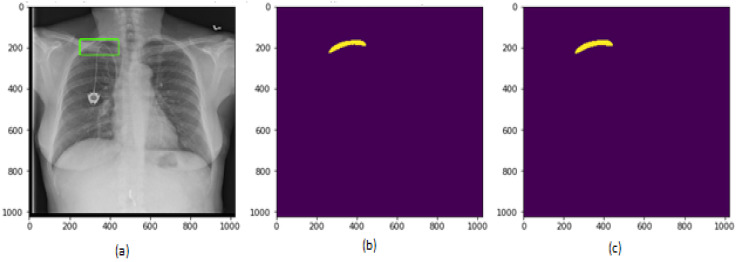
Results on test dataset for patient 1, (**a**) chest X-ray image; (**b**) segmentation mask generated by proposed model; (**c**) segmentation mask in ground truth.

**Figure 15:**
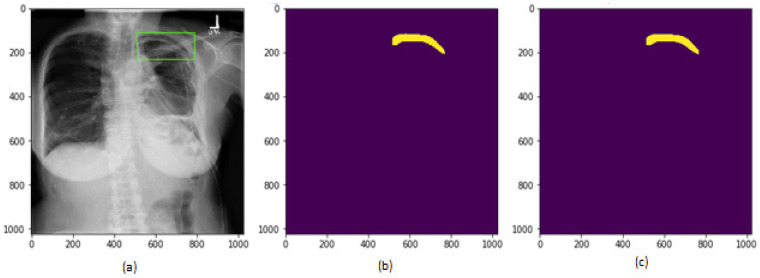
Results on test dataset for patient 2, (**a**) chest X-ray image; (**b**) segmentation mask generated by proposed model; (**c**) segmentation mask in ground truth.

After the training of the proposed model, the test images were applied to the model to generate the segmentation masks. The segmentation masks generated by the proposed model are shown in Figure 14 and Figure 15 for two different patient chest X-ray images.

### 6.2. Analysis Based on Loss Scores

The loss score of a neural network represents the prediction error of the model. A curve can be plotted to represent the loss generated by the predictions of a model. The model is designed to minimize the loss function. The performance of the proposed model was analyzed by evaluating the three different types of loss scores, as given below:

#### 6.2.1. Results for Class Loss

Class loss represents the closeness of the model to predicting the correct class. There are two classification losses in the MRCNN model.

(a) *RPN class loss* is defined as the RPN anchor classifier loss that represents the closeness of the RPN in predicting the class label.
(b) *MRCNN class loss* represents the loss due to the classifier head of the Mask RCNN.

The classification loss employed in the model is the cross entropy loss function [38]. It represents the difference in the information contained in the predicted class probability and the true class. It is defined as given in Equation (1).

(1) $$L_{\mathrm{cls}}(P_{i},{P_{i}}^{*})=-\sum_{i}P_{i}^{*}\log(P_{i})$$

where, *P_i_* is the Predicted probability of anchor *I* representing an object class and P_i_* is the ground truth label for anchor *i*, being an object. In the present work, there are two classes, background and pneumothorax, thus the formula to find class loss changes, as in Equation (2):

*L_cls_*(*P_i_*,P_i_*) = −P_i_*log*P_i_* − (1 − P_i_*)log(1 − *P_i_*) (2)

Table 7 gives the minimum RPN class loss scores and MRCNN class loss scores for the ResNet50 and ResNet101 backbones with the different learning rates and epochs. From Table 7, it can be deduced that the value of total class loss is minimal for both the learning rates in the case of the proposed model as compared to conventional models.

**Figure 16:**
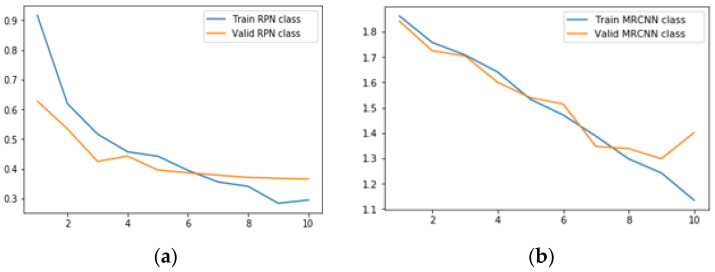
Class loss for the proposed model with LR = 0.0006, epochs = 10, (**a**) RPN class loss; (**b**) MRCNN class loss.

It is also clear from Table 7 that the minimum class loss is at the learning rate 0.0006 with 10 epochs. Hence, Figure 16 is showing the generated class loss scores plot for the proposed model for 10 epochs with a learning rate of 0.0006 only. During simulation, RPN validation class loss is constant after the sixth epoch, and MRCNN validation class loss is the least at the ninth epoch.

#### 6.2.2. Results for Bounding Box Regression Loss

The bounding box regression loss of a model represents the distance between the true box coordinates and the predicted box coordinates. There are two types of bounding box losses:

(a) RPN bbox loss provides the RPN bounding box loss values reflecting the distance between the true boxes coordinates and the predicted RPN boxes coordinates.
(b) MRCNN bbox loss provides the MRCNN bounding box loss values reflecting the distance between the true boxes coordinates and the predicted MRCNN coordinates. Smooth L1 loss [37,38] is used to represent bounding box regression as shown in Equations (3) and (4).
  (3) $$L_{box}(P_{i},t_{i})=\frac{\lambda }{N_{box}}\sum_{i}P_{i}^{*}L_{1}^{smooth}(t_{i}^{}-t_{i}^{*})$$
  Here, *λ* represents the balancing parameter set to 10.
  *N_bo_*_x_ is the normalization term equal to the number of anchor locations, set to 256.
  *P_i_* represents the predicted probability that anchor *i* is an object.
  P_i_* L_1_ shows that regression loss is active for positive anchors (P_i_* = 1) only.
  *t_i_* represents the predicted four coordinates.
  t_i_* represents ground truth coordinates.
  (4) $$L_{1}^{smooth}=0.5(Y_{\mathrm{true}}-Y_{\mathrm{pred}}{)}^{2}\;\mathrm{if}\;|x|\;<\;1\;\;=\;|Y_{\mathrm{true}}-Y_{\mathrm{pred}}|-0.5\;\mathrm{otherwise}$$

To compute this loss, the algorithm first finds the absolute difference between the true and predicted values, (*Y_true_* − *Y_pred_*). It then checks if (*Y_true_* − *Y_pred_*) is less than one or not. It further computes $L_{1}^{smooth}$. The total regression loss is computed using the formula given in Equation (3).

Table 8 gives the minimum RPN bbox Loss scores and MRCNN bbox Loss scores for the ResNet50 and ResNet101 backbones with the different learning rates and epochs. From Table 8, it can be seen that the value of total bbox loss is minimal for both the learning rates in the case of the proposed model as compared to conventional models. The minimum RPN bbox loss is observed with ResNet 50 as a backbone with LR 0.0006 and 10 epochs whereas MRCNN bbox loss is the least with the ResNet101 as a backbone with LR0.0006 and 10 epochs.

**Figure 17:**
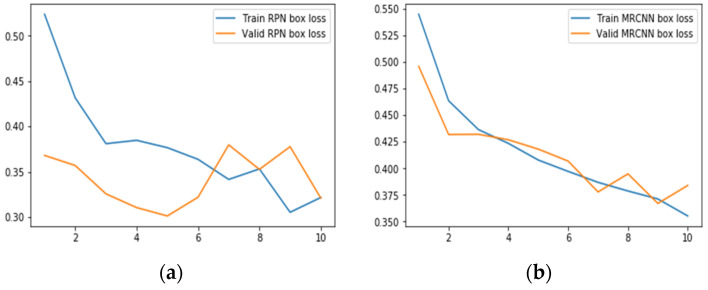
Bounding Box Regression loss for proposed Model with LR = 0.0006, Epochs = 10, (**a**) RPN bbox loss; (**b**) MRCNN bbox loss.

Figure 17 shows the generated bounding box loss plot for the proposed model simulated for 10 epochs with a learning rate of 0.0006. In Figure 17a, at 10th epoch, the RPN train box and validation box losses are the same. In Figure 17b, MRCNN validation box losses are fluctuating.

#### 6.2.3. Results for Mask Loss

Mask loss is the mean binary cross-entropy loss for the masks head [45,46]. It is defined in Equation (5):

(5) Lmask=−1m∑1≤ij≤m[yijlogyij^K+(1−yij)log(1−yij^K)]

where, $y_{ij}$ represents the label given to cell (*i*, *j*) in the ground truth mask; yij^k represents the label predicted for the same cell in the mask generated by the model.

Table 9 lists the minimum mask loss scores for ResNet50 and ResNet101 with the different learning rates and epochs. The MRCNN mask loss is the least with ResNet101 as the backbone with LR 0.0006 and 10 epochs.

**Figure 18:**
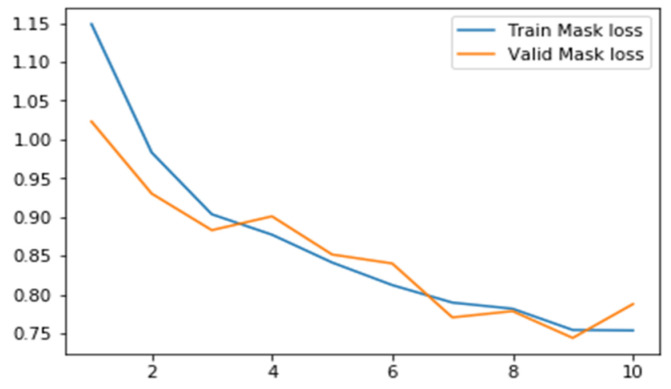
Mask loss scores for the proposed model with LR 0.0006, Epochs = 10.

Figure 18 represents the mask loss for training and validation loss for the proposed model; the validation mask loss is fluctuating.

#### 6.2.4. Results for Total Loss

The total loss in the MRCNN model is the sum of class loss, bounding box regression loss, and the mask loss as given in Equation (6).

*Total loss*, *L_total_* = *L_rpncls_* + *L_mrcnncls_* + *L_rpnbbox_* + *L_mrcnnbbox_* + *L_mask_* (6)

where, *L_rpncls_* = RPN class loss, *L_mrcnncls_* = MRCNN class loss, *L_rpnbbox_* = RPN bounding box loss, *L_mrcnnbbox_* = MRCNN bounding box loss, and *L_mask_* = mask loss.

Table 10 gives the total loss score for the ResNet50 and ResNet101 backbones with the different learning rates and epochs. From the results shown in Table 10, it was interpreted that the proposed model has minimum loss scores with ResNet101 as the backbone and an LR of 0.0006 simulated for 10 epochs.

**Figure 19:**
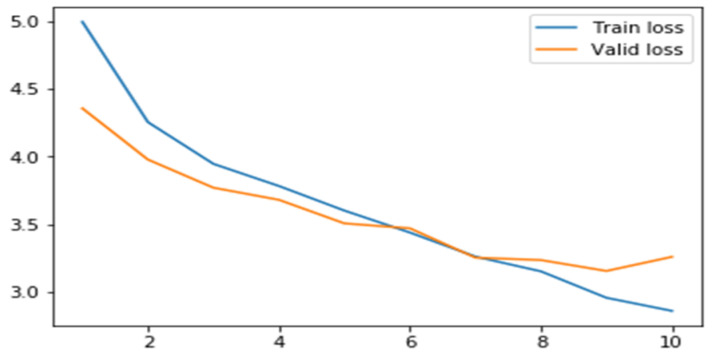
Total loss for the proposed model with an LR 0.0006 and 10 epochs.

Figure 19 represents the generated total loss scores plot for the proposed model with ResNet101 as a backbone FPN, simulated for 10 epochs with a learning rate of 0.006. The plot shows that overall validation loss for the proposed model is higher than the training loss.

#### 6.2.5. Analysis of the Proposed Model for All the Losses

**Figure 20:**
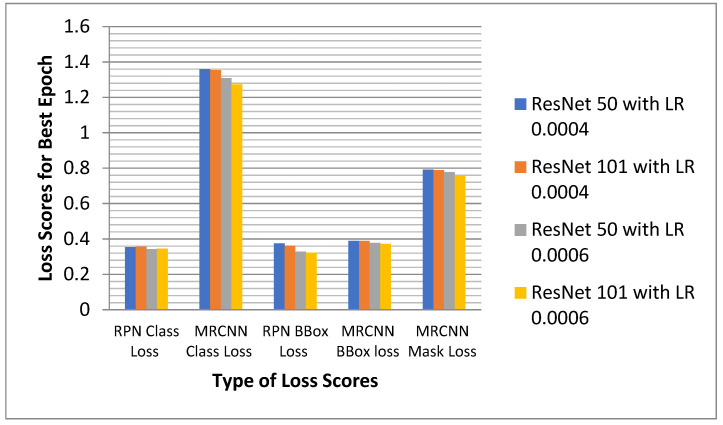
Comparative Analysis of ResNet101 and ResNet50 FPN for all type of losses.

The proposed model and conventional model were simulated for two different learning rates, 0.006 and 0.0004, with two different epochs of 10 and 12. The loss scores for the model were generated after the execution of all the epochs. The best epoch was selected based on the generated scores. The proposed MRCNN model with ResNet101 asa backbone has been compared with MRCNN with ResNet50 as a backbone. Figure 20 compares the total loss values for the two models simulated with LR 0.0006 and LR 0.004. From Figure 20, it was observed that the ResNet101 backbone model with a learning rate of 0.0006 has a minimum loss of 3.075138, which is highlighted in purple. The Resnet101 shows minimum RPN class loss, minimum MRCNN class loss, minimum RPN bbox loss, minimum MRCNN bbox loss, and MRCNN mask loss for a learning rate of 0.0006.

### 6.3. Comparison with Existing Models

The proposed Mask RCNN model based on ResNet101 as a backbone FPN was used to localize the regions containing pneumothorax automatically on the chest X-ray images. The proposed model was also evaluated on the basis of IoU [47]. This defines the amount of intersecting area between the predicted mask segment and the ground truth mask segment, divided by the total area of union between the predicted mask segment and the ground truth mask (Equation (7)).

(7) $$\mathrm{IoU}=\frac{|A\cap B|}{|A\cup B|}$$

where, A is the ground truth mask segment; B is the predicted mask segment.

Our proposed model produced an IoU of 0.829 (at LR = 0.0006). The IoU of the proposed model based on ResNet101 is higher as compared to the model based on ResNet50. Table 11 compares the performance of the proposed model with existing models.

The proposed Mask RCNN with ResNet101 as a backbone performed better than the existing models, as shown in in Table 11.

However, the deep learning models suffered from over-fitting and parameter tuning problems. Additionally, these models generally require image filters to remove the impact of noise from images to achieve better results. Therefore, in the near future, we will use metaheuristics techniques to tune the proposed model [49]. Additionally, various filters such as a gain gradient image filter [50] or notch-based filter [51] were used to filter the imaging datasets.

## 7. Conclusions and Future Scope

Deep learning algorithms help the machines to interpret the images. The advancement in the field of AI-based image processing has opened an extensive range of opportunities in the area of medical disease diagnosis and prognosis. We proposed a Mask RCNN model with transfer learning for automatic segmentation of pneumothorax in chest X-ray images. The proposed model used ResNet101 as a feature pyramid network. The proposed model was compared with the conventional model utilizing ResNet50 as an FPN. Both the models were trained on an SIIM-ACR pneumothorax dataset available at Kaggle. The models were simulated with two different learning rates of 0.0006 and 0.0004 and two different epochs values of 10 and 12. The simulation results demonstrate that the proposed model with ResNet101 as an FPN has better performance as compared with the conventional model with ResNet50 as an FPN.

The Mask RCNN model employed in the present work is based on instance segmentation. As discussed in the previous section, it has certain limitations while working on the edges of the image. Therefore, there are many different semantic image segmentation models such as UNet, DeepLab, etc. that can be used for segmentation of pneumothorax in chest X-ray images. The future work will use these models for pneumothorax segmentation to achieve higher accuracy. These deep learning models that are capable of generating automatic segmentation of pneumothorax on CXR images will benefit the health department by providing early diagnosis of the disease and clear insight into the geometric size of the abnormality. It can help doctors in taking crucial decisions regarding the medication.

## Figures and Tables

**Table 1:** Differences between ResNet50 and ResNet101 layers.

Layer Name	ResNet50	Resnet101
Convolution 1	7 × 7, 64, stride 2	
Convolution 2×	3 × 3 Max pool, stride 2	
	$\left[\begin{array}{cc} 1\times 1 & 64\\ 2\times 3 & 64\\ 1\times 1 & 256 \end{array}\right]\times 3$	$\left[\begin{array}{cc} 1\times 1 & 64\\ 2\times 3 & 64\\ 1\times 1 & 256 \end{array}\right]\times 3$
Convolution 3×	$\left[\begin{array}{cc} 1\times 1 & 128\\ 3\times 3 & 128\\ 1\times 1 & 512 \end{array}\right]\times 4$	$\left[\begin{array}{cc} 1\times 1 & 128\\ 3\times 3 & 128\\ 1\times 1 & 512 \end{array}\right]\times 4$
Convolution 4×	$\left[\begin{array}{cc} 1\times 1 & 256\\ 3\times 3 & 256\\ 1\times 1 & 1024 \end{array}\right]\times 6$	$\left[\begin{array}{cc} 1\times 1 & 256\\ 3\times 3 & 256\\ 1\times 1 & 1024 \end{array}\right]\times 23$
Convolution 5×	$\left[\begin{array}{cc} 1\times 1 & 512\\ 3\times 3 & 512\\ 1\times 1 & 2048 \end{array}\right]\times 3$	$\left[\begin{array}{cc} 1\times 1 & 512\\ 3\times 3 & 512\\ 1\times 1 & 2048 \end{array}\right]\times 3$
	Average pool, 1000-d FC, softmax	

**Table 2:** RPN parameters.

S. No.	Simulation Parameter	Parameter Value
1	RPN anchors per image	256
2	Anchor areas	32^2^, 64^2^, 128^2^, 256^2^
3	Anchor ratios	0.5, 1, 2
4	Anchor stride	1
5	RPN NMS Threshold	0.7

**Table 3:** Training, validation, and testing dataset (split).

Number of	Before Sampling	After Sampling
Training Images	10,675	15,629
Validation Images	—	1320
Test Images	1377	1377
Images with Pneumothorax	2379	8316
Percentage of Positive Cases (%)	22.29	53.21

**Table 4:** Pixel positions and length of pixels having values ‘0’ and ‘1’for mask generation for reference image ID.

Initial Pixel Position	Length of Pixels Having Value ‘1’	Final Pixel Position	Length of Pixels Having Value ‘0’
759,441	11	759,452	1010
760,462	15	760,477	1007
761,484	18	761,502	1005
762,507	19	762,526	1005

**Table 5:** Experimental parameter values.

S. No.	Simulation Parameter Name	Parameter Value	S. No.	Simulation Parameter Name	Parameter Value
1	Number of classes	2	7	GPU count	1
2	Image dimension	512 × 512	8	Images per GPU	11
3	RPN anchor scales	32,64,128,256	9	Weight Decay	0.0005
4	Train RoIs per image	32	10	Learning momentum	0.9
5	NMS threshold	0.1	11	Steps per epoch	350
6	Batch size	11	12	Validation Steps	120

**Table 6:** Simulation parameters for stage 1 and stage 2 training.

Sr. No.	Simulation Parameters	Parameter Value for Stage 1	Parameter Value for Stage 2
1	Learning rate	0.0012	0.0006
		0.0008	0.0004
2	Number of epochs	1	10, 12
3	Layers trained	heads	all
4	Augmentation	None	yes

**Table 7:** Class loss values for validation data with different learning rates and epochs.

Model	Backbone Network	Learning Rate	Total Epochs	Minimum RPN Class Loss	Minimum MRCNN Class Loss	Total Class Loss = RPN Class Loss + MRCNN Class Loss
Conventional Model	ResNet50	0.0004	12	0.353907	1.359199	1.713106
Proposed Model	ResNet101		12	0.355985	1.353964	1.709949
Conventional Model	ResNet50	0.0006	10	0.343072	1.309230	1.652302
Proposed Model	Resnet101		10	0.345099	1.273622	1.618721

**Table 8:** BBox loss values for validation data with different learning rates and epochs.

Model	Backbone Network	Learning Rate	Total Epochs	Minimum RPN BBox Loss	Minimum MRCNN BBox Loss	Total Bbox Loss = RPN BBox Loss + MRCNN BBox Loss
Conventional Model	ResNet50	0.0004	12	0.374971	0.389217	0.764188
Proposed Model	ResNet101		12	0.362586	0.388650	0.751236
Conventional Model	ResNet50	0.0006	10	0.327884	0.378461	0.706345
Proposed Model	Resnet101		10	0.323304	0.372548	0.645852

**Table 9:** MRCNN mask loss for validation data with different learning rates and epochs.

Model	Backbone Network	Learning Rate	Total Epochs	Minimum MRCNN Mask Loss
Conventional Model	ResNet50	0.0004	12	0.791617
Proposed Model	ResNet101		12	0.788237
Conventional Model	ResNet50	0.0006	10	0.777462
Proposed Model	Resnet101		10	0.760439

**Table 10:** Total loss scores for validation data with different learning rates and epochs.

Model	Backbone Network	Learning Rate	Total Epochs	Minimum Total Loss	Model Training Time
Conventional Model	ResNet50	0.0004	12	3.268911	31,380 s
Proposed Model	ResNet101		12	3.249549	30,430 s
Conventional Model	ResNet50	0.0006	10	3.136109	28,800 s
Proposed Model	Resnet101		10	3.075138	27,640 s

**Table 11:** Comparison with existing models on the basis of IoU.

Work	Model	Backbone	IoU
Hongyu et al. [29]	Mask RCNN	ResNet50	0.81
Ayat Abdella et al. [30]	U-Net	ResNet34	0.7822
Jakhar et al. [48]	U-Net	ResNet50	0.826
Ours	Mask RCNN	ResNet101	0.829

## Data Availability

Publicly available datasets were analyzed in this study. This data can be found here: https://www.kaggle.com/jesperdramsch/siim-acr-pneumothorax-segmentation-data (accessed on 18 January 2022).
